# Gut Microbiota-Derived Mediators as Potential Markers in Nonalcoholic Fatty Liver Disease

**DOI:** 10.1155/2019/8507583

**Published:** 2019-01-02

**Authors:** Gemma Aragonès, Sergio González-García, Carmen Aguilar, Cristóbal Richart, Teresa Auguet

**Affiliations:** ^1^Grup de Recerca GEMMAIR (AGAUR)-Medicina Aplicada, Departament de Medicina i Cirurgia, Universitat Rovira i Virgili (URV), Institut d'Investigació Sanitària Pere Virgili (IISPV), 43007 Tarragona, Spain; ^2^Servei Medicina Interna, Hospital Universitari Joan XXIII Tarragona. Mallafré Guasch, No. 4, 43007 Tarragona, Spain

## Abstract

Nonalcoholic fatty liver disease (NAFLD) is a common, multifactorial, and poorly understood liver disease whose incidence is globally rising. During the past decade, several lines of evidence suggest that dysbiosis of intestinal microbiome represents an important factor contributing to NAFLD occurrence and its progression into NASH. The mechanisms that associate dysbiosis with NAFLD include changes in microbiota-derived mediators, deregulation of the gut endothelial barrier, translocation of mediators of dysbiosis, and hepatic inflammation. Changes in short chain fatty acids, bile acids, bacterial components, choline, and ethanol are the result of altered intestinal microbiota. We perform a narrative review of the previously published evidence and discuss the use of gut microbiota-derived mediators as potential markers in NAFLD.

## 1. Introduction

Nonalcoholic fatty liver disease (NAFLD) comprises a variety of diseases extending from simple steatosis (SS), nonalcoholic steatohepatitis (NASH), fibrosis, and cirrhosis, with a growing prevalence worldwide, reaching around 30% of global population [1]. NASH is the severe form of the disease, and patients could develop liver cirrhosis and hepatocellular carcinoma with aging. NASH is characterized by the presence of hepatocyte ballooning and inflammation, with a worldwide prevalence of 2–3%; however almost one-third of NAFLD affected subjects progress to NASH [1].

Precise histological diagnosis, including disease stages (SS and NASH), is commonly based on liver biopsy [2]; however, biopsy comprises several potential problems such as bleeding, abdominal pain, and needs to be performed in a special clinical setting under expertise supervision [3]. Thus, there is a need for reliable and cost-effective noninvasive biomarkers, to avoid the invasiveness of biopsy. However, until date, none of the previously explored surrogate blood markers have been confirmed in large cohorts of biopsy-proven NAFLD or have proper specificity for NASH diagnosis.

Previous evidence has linked intestinal microbiota dysbiosis with obesity, insulin resistance, metabolic syndrome, and NAFLD [4–6]. Due to dysbiosis, the permeability of intestinal barrier is compromised and substances such as short-chain fatty acids, bile acids, bacterial components, choline, and endogenous ethanol reach the liver which seem to contribute to the pathogenesis of NAFLD. More recently, other metabolites or proteins as angiopoietin-like protein 4 (ANGPTL4), resistin-like molecule *β* (RELM *β*), neurotensin, glucagon-like peptide-1 (GLP-1), glucagon-like peptide-2 (GLP-2), and fibroblast growth factor 19 (FGF19) have been suggested to be involved in NAFLD pathogenesis [7, 8]. It is important to note that some of these metabolites may be employed as potential markers of NAFLD occurrence and progression.

In order to give a broad overview of primary literature published on this topic, we have used narrative review as literature search strategy in the present article. In this sense, this narrative review will discuss NAFLD and (a) gut microbiome dysbiosis and (b) main gut microbiota-derived mediators. Their potential use as biomarkers for evaluating the status of NAFLD will also be briefly discussed.

## 2. Gut Microbiome Dysbiosis and NAFLD

Intestinal microbiome is composed mainly of bacteria, virus, and fungi, with several functions, such as host nutrition, bone mineralization, immune system regulation, xenobiotics metabolism, proliferation of intestinal cells, and protection against pathogens [9, 10]. The microbiome is specific to an individual and highly resilient to changes. However, it can be affected by several factors, intrinsic and extrinsic to the hosts, such as subject's genetic, dietary habits, antibiotics, and environmental changes [11–13]. Gut microbiota comprises about 1000 different species, but Firmicutes and Bacteroidetes are the most important phyla in intestinal bacteria, with a proportion of over 90% of the total [14]. A disruption in the composition—quantitative or qualitative—of the normal microbiota, is known as gut dysbiosis [15, 16]. Generally, this process includes an unfavorable change in the bacterial composition with a reduction in autochthonous (Firmicutes) bacteria and growth of other taxa (Bacteroidetes, Actinobacteria) [17].

Dysbiosis may adversely impact metabolism and immune responses, favoring NAFLD and NASH. Because of gut dysbiosis, there is an elevated production of toxic bacterial components and metabolic mediators, which consequently accumulate in the intestine. In addition, an increase in intestinal permeability and further disruption of the epithelial barrier lead to the efflux of these gut microbiota-derived mediators [16], which could reach the liver through portal circulation favoring hepatic inflammation and the development of NAFLD [18, 19]. Approximately, 70-75 % of blood that reaches the liver comes from the portal vein, which drains blood from mesenteric veins of the intestinal tract [20]; and, after the disruption of the intestinal epithelial-barrier, the liver is exposed to the microbial products and metabolites resulting from the metabolism of bacteria [21, 22]. In this sense, it has been demonstrated that patients with NAFLD have dysbiosis of intestinal microbiota, gut epithelial barrier dysfunction, and increased translocation of bacterial components to the liver [23].

Instead of the evidence relating disruption of the gut-barrier and hepatic diseases [21, 22], previous studies have demonstrated that increased intestinal permeability and endotoxin levels are not present in all patients that develop NAFLD [24, 25]. Therefore, intestinal barrier dysfunction with subsequent translocation of bacterial components because of dysbiosis is not the hallmark in the development or progression of the disease. For this reason, other mediators derived from gut microbiota dysbiosis might be also related to the pathogenesis of the disease. These mediators could be metabolites due to metabolic changes related to microbiome dysbiosis.

Several previous studies in clinical settings have associated intestinal dysbiosis with the occurrence of NAFLD [26–28] and with the progression to NASH [29, 30]. Gut microbiota-derived mediators—metabolites and bacterial components—resulting from gut dysbiosis could be representative of NAFLD progression through several mechanisms: (1) enhanced energy extraction from food nutrients by formation of short-chain fatty acids; (2) modulation of bile acid synthesis which are crucial for fat absorption and affect metabolism of glucose via farsenoid X receptor; (3) innate-immune system activation by bacterial components translocation; (4) endogenous ethanol production; and (5) reduction of choline metabolism which reduces efflux of VLDL from hepatocytes promoting inflammation [15] among others. These mechanisms involve translocation of both microbial degradation products and microbiota-derived metabolites such as short-chain fatty acids, bile acids, ethanol, and choline, which may be potentially evaluated as noninvasive blood markers of NAFLD progression.

## 3. Gut Microbiota-Derived Mediators in NAFLD

In the present section we will focus on the main gut microbiota-derived mediators related to NAFLD: short-chain fatty acids, bile acids, bacterial components, endogenous ethanol, and choline deficiency. Also, we have performed a summarizing table (Table 1) including the main published human studies.

### 3.1. Short-Chain Fatty Acids

Short-chain fatty acids (SCFAs), such as acid acetic, acid propionic, and acid butyric, are molecules with seven carbon atoms or less, mainly produced by the fermentation of indigestible carbohydrate by gut microbiota [31]. In general, these SCFAs have several effects on energy metabolism, immune response, and adipose tissue expansion and act as signaling molecules between the gut microbiota and the subject [31, 32]. Not only do SCFAs provide important sources of nutrients and energy from the intestinal epithelium but also they are precursors for lipogenesis and gluconeogenesis [32].

In general, changes in the microbiota result in increased production of SCFA in the intestine with an increased transport of monosaccharides to the liver, promoting hepatic lipogenesis and steatosis [47]. Increased acetate in the liver causes accumulation of triglyceride, because it is an important substrate for fatty acid synthesis [48], whereas raised levels of propionate promote liver gluconeogenesis [49].

Experimental studies have demonstrated that these SCFAs can remodel regulatory T cell expansion and enhance neutrophil chemotaxis, modulating inflammation in mice models [47–50]. Also, SCFAs modulate the production of several inflammatory cytokines, including tumor necrosis factor-*α* (TNF-*α*), interleukin-2 (IL-2), IL-6, and IL-10 [51]. Recently, some studies found that high concentrations of intestinal SCFAs as result of dysbiosis and their G protein-coupled receptors play an important key role in NAFLD progression [52, 53]. SCFAs activate G-protein coupled receptors (GPCRs), specifically the subtypes GPR41 and GPR43. Activation of these GPCRs stimulates secretion of peptide-YY, inhibits gut motility, and slows intestinal transit. Therefore, nutrient absorption and energy harvest from the diet increase, promoting hepatic lipogenesis [54, 55]. Additionally, activation of GPR41 and GPR43 promotes secretion of glucagon-like-peptide-1 (GLP-1), which activates genes in hepatocytes that regulate fatty acid *β*-oxidation and insulin sensitivity [55, 56], promoting NAFLD occurrence and progression.

However, other previously published studies point in another direction and have reported that SCFAs could be beneficial in the progression of NAFLD; for example, butyrate activates AMP-activated protein kinase (AMPK) in the liver [57] and accelerated the assembly of tight junction proteins in the colonic epithelial cell line Caco-2 [58], improving intestinal barrier dysfunction. In addition, butyrate is able to modulate epigenetic changes decreasing the activity of histone deacetylases (HDACs), which further increase in the number of regulatory T cells, suppressing the immune response and reducing liver inflammation [59].

Furthermore, clinical studies have demonstrated SCFA enrichment in fecal samples of children and adults with NAFLD [33, 34]. These results confirm the relation between excretion of SCFA and NAFLD, although there are differences in relation to the SCFA concentrations excreted which could be related to differences in the age of subjects, diet, environmental factors, and technical issues, related to the volatility of the SCFAs [23].

The close relation between microbiota dysbiosis and SFCAs production—as part of carbohydrate bacterial fermentation—with the results of previous experimental and clinical studies provide evidence of their potential use as markers of NAFLD progression.

### 3.2. Bile Acids

Bile acids (BA) are steroidal molecules synthesized after cholesterol oxidation by enzymes presented in hepatocytes which are important in the regulation of glucose and lipid metabolism. They participate in the digestion and solubilization of lipids and regulate hepatic glucose and inflammation. Also, they are able to control their own synthesis through the activation of farnesoid X receptor (FXR) [60, 61]. In addition, BA function as signaling molecules that modulate several physiological processes, and gut dysbiosis can change BA pool characteristics through their effects on BA metabolism [61–63].

Gut microbiota is a critical modulator of BA pool size and composition and the process of dysbiosis could substantially alter systemic concentrations of conjugated and/or secondary bile acids, as well as increasing their synthesis. An increased level of BA causes an activation of cell death pathway mediated by inflammatory and oxidative stress cascades in liver tissue [64, 65]. In turn, BA can have direct effects on intestinal microbiota by causing membrane disruption through their amphipathic properties, acting as a detergent for cellular membranes. This increased intestinal permeability, associated with BA modifications, has been linked to metabolic endotoxemia, insulin resistance, and inflammatory cytokine release with enhanced proinflammatory signaling cascades, common findings in patients with NAFLD [66, 67].

Previous investigations have demonstrated a BA increase in biological fluids of patients with NASH compared to subjects with healthy livers and an evident association with intestinal dysbiosis [35–37]. Kalhan et al. performed a metabolomic profile of derivates from bile acid metabolism, glutathione metabolism, lipids, carbohydrate, and amino acids, which do not differentiate patients with steatosis from those with steatohepatitis. However it revealed significant changes in certain metabolic pathways, suggesting that a metabolome study of BA and derivates could potentially be used as a noninvasive marker to evaluate the status of NAFLD and the therapeutic patient's outcome [37]. Also, levels of BA have been correlated with histopathological features, such as the degree of hepatic steatosis, the presence of cellular ballooning, and the severity of fibrosis in patients with NASH [38]. Ferslew et al. reported that NASH patients have higher total serum BA concentrations than healthy volunteers, specifically increase in taurine- and glycine-conjugated primary and secondary BA, under fasting and postprandial conditions, confirming the disruption in bile acid homeostasis in NASH physiopathology [35]. In addition, plasma levels of glycocholate, taurocholate, glycochenodeoxycholate, taurochenodeoxycholate, and ursodeoxycholic acid were increased in patients with NASH compared with patients with SS [68]. Also, levels of taurolithocholic acid, glycocholate, and taurocholate have been correlated with severity of portal inflammation, lobular inflammation, steatosis, and hepatocyte ballooning, respectively [68].

In children with NAFLD, changes in circulating BA profile have been reported too [39, 69]. The research of Jahnel et al. demonstrates that serum BA levels decrease in early NAFLD and increase during progression to fibrosis in obese children. These authors postulated that BA may have a value as a noninvasive biomarker in pediatric NAFLD progression [69].

Experimental studies have demonstrated that dysbiosis of the gut microbiota can modulate the activity of FXR in the intestine, affecting as consequence lipid metabolism in the liver [4]. Specifically, FXR not only plays an important role in maintaining bile acids but also regulates glucose and lipid metabolism via different mechanisms, such as increasing insulin sensitivity, repressing hepatic gluconeogenic genes, and increasing hepatic glycogen synthesis [70, 71].

Considering the numerous experimental and clinical published studies associating gut dysbiosis, bile acids, and NAFLD, it is expected that these molecules could be proposed as potential noninvasive markers of the disease, specifically the secondary bile acids deoxycholic acid (DCA) and lithocholic acid (LCA), which cannot be produced without bacterial fermentation [72].

### 3.3. Bacterial Components

The liver is exposed to potentially harmful substances derived from the gut, considered as pathogen-associated molecular patterns (PAMPs), including translocated bacteria, lipopolysaccharide (LPS), DNA, RNA, and endotoxins, which are potent inducers of tissue inflammation [15, 73]. These PAMPs might contribute to the pathogenesis of fatty liver disease by activation of the innate immune system via toll-like receptors (TLRs), which recognize these gut-derived bacterial components [73]. The translocation of these bacterial components from the gut into the portal system is facilitated by the disruption in tight junctions, which normally seal the junction between intestinal endothelial cells at their apical border, facilitated by gut microbiome dysbiosis [73].

There is evidence that dysbiosis causes permeability changes that increase portal levels of gut-derived TLR ligands (LPS or endotoxin), which further activate TLR4 on hepatic Kupffer and stellate cells [74]. During receptor activation, the adaptor molecule myeloid differentiation factor 88 (MyD88) is activated, and the downstream signaling MyD88-dependent pathway results in the activation of the nuclear factor-K*β* (NF-K*β*) leading to the expression of proinflammatory cytokines (TNF-*α*, IL-6, IL-8, and IL-12) and chemokines (interferon-*γ* [IFN-*γ*] and monocytes chemotactic protein-1 [MCP-1]), promoting inflammation [52, 74]. There are several intracellular cascades involved in this process which include stress-activated and mitogen-activated protein kinases, JNK (c-Jun N-terminal kinase) and p38 mitogen-activated kinases, which triggers transcription of proinflammatory genes and facilitates hepatic migration of neutrophils and monocytes, generation of oxidative stress mediators—nitrogen and oxygen reactive species—low-grade systemic inflammation, and hepatic injury [75].

In addition, TLR signaling, as a result of gut dysbiosis, can also lead to the production of inflammasomes, in peripheral and parenchymal cells, which activate a variety of processes, including cleavage of procaspase-1 to form active caspase-1, resulting in cell death dependent on caspase-1 and caspase-3 [76]. Inflammasome, which is a multimeric signaling platform that leads to the production of IL-18 and IL-1*β*, through NRLP3 (NOD-like receptors, pyrin domain containing 3) and NRLP6, is activated by LPS derived from dysbiosis of gut microbiota via TLR4 and TLR9 response. Reports have associated inflammasome activation with the development of liver steatosis, inflammation, and fibrosis in NAFLD patients [77, 78].

Previous studies have demonstrated that endotoxemia markers, as a result of gut dysbiosis, were associated with the pathogenesis and severity of NAFLD [79, 80]. In addition, other studies have established that the increase in endotoxin level is related to IL-1*α* and TNF-*α* production [81, 82]. In patients with NAFLD gut permeability and the prevalence of small intestinal bacterial overgrowth have been associated with the severity of steatosis [66]. In biopsy-proven human NASH, plasma IgG levels against endotoxin were found to be increased with NASH grade severity, suggesting the deleterious effect of chronic endotoxin exposure [83]. Also, enhanced expression of TLR4, the release of IL-8, and high levels of LPS have been demonstrated in NAFLD patients [40, 67]. Furthermore, two recent studies in obese children with NAFLD showed that intestinal permeability was correlated with the degree of hepatic damage and endotoxin levels. In addition, urinary metabolome analyses identified metabolite changes associated with dietary habits, intestinal permeability, and small intestinal bacterial overgrowth (SIBO) [84, 85]. However, other reports did not reveal an association between endotoxemia and NAFLD/NASH development, suggesting that endotoxemia may not be the only driver of disease progression in all patients [41].

Multiple experimental studies have demonstrated that a high-fat diet can increase the proportion of LPS derived from gut bacteria and administration of endotoxin has been shown to induce insulin resistance and weight gain [86, 87]. On the other hand, some authors have proposed recently that the small intestine shields the liver from otherwise toxic fructose exposure, via gut microbiota [88].

There is an evident relation between gut dysbiosis, bacterial-derived components, inflammatory response, and NAFLD; therefore these bacterial mediators, especially circulating TLRs, might be used as potential noninvasive markers of disease progression.

### 3.4. Endogenous Ethanol Production

Dysbiosis due to changes in microbiome composition profile, specifically in* Escherichia coli* and other* Enterobacteriaceae*, increases endogenous ethanol production [41], which might contribute to liver injury by affecting intestinal permeability, with disruption of intestinal tight junctions. This allows endotoxins and ethanol trigger TLR response and inflammasome activation, which further inflammatory response in liver tissue [89]. In addition to the proinflammatory response, ethanol promotes oxidative damage and hepatocyte necrosis because of the formation of reactive oxygen and nitrogen species [90]. Endogenous ethanol inhibits the tricarboxylic acid cycle, thus increasing levels of acetate, thereby promoting triglyceride accumulation in hepatocytes [48]. Ethanol can also increase the activity of the enzyme cytochrome P450 2E1 (CYP2E1) [91] which catalyze the oxidation of ethanol but produce free radicals favoring oxidative damage, mitochondrial dysfunction and liver inflammation [90, 92].

Several studies have detected increased levels in nondietary ethanol, derived from bacteria, in obese patients [41, 93], and in patients with NASH [41, 42, 94], with a related upregulation of hepatic alcohol metabolizing capacity (alcohol dehydrogenase, aldehyde dehydrogenase, and cytochrome P450 2E1) [94]. In this sense, the group of Zhu et al. propose that microbiomes rich in ethanol-producing* Escherichia* may be a risk factor for progression from obesity to NAFLD [41]. Besides* Escherichia coli*, other gut microbial genera, including* Bacteroides*,* Bifidobacterium*, and* Clostridium*, can produce alcohol and generate a significant ethanol-mediated damage [41]. So, production of endogenous ethanol by the gut microbiota may act as a hepatotoxin, contributing to the development of NAFLD and its progression to NASH [95]. In addition, children with fatty liver showed higher levels of endogenous ethanol and LPS related to gut microbiome [43]. Moreover, children with NASH had higher serum levels of ethanol than obese and healthy children without NASH [41, 42], confirming that endogenous ethanol might contribute to the pathogenesis of NAFLD and NASH.

Gut dysbiosis with the increase in ethanol-producing bacteria (*Enterobacteriaceae*) in the microbiome is the main hypothesis to explain the differences in blood ethanol in NAFLD patients, and the importance of ethanol-derived microbiome in NASH [85]. However, other hypotheses suggest that alterations in insulin signaling followed by decreased alcohol dehydrogenase activity in the liver could be responsible for an impaired ethanol metabolism [42].

In summary, the proinflammatory and prooxidative damage as a result of endogenous ethanol in the liver, which might contribute to the pathogenesis of NAFLD, has been demonstrated; and the previous reports may support its use as a noninvasive marker of disease progression.

### 3.5. Reduction of Choline Metabolism

Choline is an essential nutrient obtained through both dietary intake and endogenous synthesis, being an important constituent of membrane phospholipids. The human gut microbiome actively metabolizes dietary components, including choline, and dysbiosis may alter its cellular disponibility and predispose the body to a deficiency of choline. Alterations in choline and phosphatidylcholine metabolism may have an impact on several physiological pathways, which could induce NAFLD. Choline deficiency prevents synthesis and excretion of very-low density lipoprotein (VLDL), leading to hepatic triglyceride accumulation and liver steatosis [44, 96]. In fact, the link between choline deficiency and accumulation of hepatic lipids has been recognized for more than 50 years [97], leading to the establishment of choline-deficient diets to induce models of NAFLD in animals.

In addition, choline can be metabolized to its derivate trimethylamine (TMA) by the intestinal microbiota. TMA reaches the liver via portal circulation and is subsequently oxidized by hepatic flavin-containing monooxygenases in the liver, forming trimethylamine-N-oxide (TMAO), which is then released into blood circulation [98, 99]. Previous studies have revealed that TMAO may affect lipid absorption and cholesterol homeostasis and modulate glucose and lipid metabolism by decreasing the total bile acid pool size [96]. The metabolism of choline to TMA induced by dysbiosis may result in reduced choline bioavailability and increased susceptibility to NAFLD [99]. TMAO modulates glucose metabolism and increases insulin resistance in mice on an HFD [100]. In addition, TMAO promotes inflammation in adipose tissue, which can induce insulin resistance by increasing the serum level of inflammatory cytokine C-C motif chemokine ligand 2 [100]. TMAO also affects lipid absorption and cholesterol homeostasis by reducing the conversion of cholesterol into bile acids [96].

A few studies have examined the association of choline with the fatty liver disease in animals and humans. A small number of human studies have shown that the consumption of a low-choline diet promotes fatty liver and liver damage [44, 101]. Other studies have pointed out that plasma free choline levels are positively related to the severity of liver steatosis, fibrosis and NASH [45, 46]. Also, a study demonstrates the presence of a low phosphatidylcholine/phosphatidylethanolamine ratio in NASH patients, in comparison to healthy subjects [102].

On the other hand, the metabolite TMAO has been associated with the occurrence of NAFLD, and TMAO raised levels correlate with the severity of steatosis, and it has been proposed as an independent risk marker for the disease [45]. The increased risk of NAFLD might be caused by TMAO due to its effect on decreasing the total bile acid pool size via several pathophysiological mechanisms [96]: (1) by decreasing the synthesis of bile acids due to the inhibition of the key enzymes CYP7A1 and CYP27A1 and (2) by limiting the enterohepatic circulation of bile acids between the liver and intestines due to the repression of multidrug resistance protein expression.

In summary, the evidence demonstrated that choline and TMAO are associated with progression of NAFLD, indicating the potential use of these gut-derived mediators of dysbiosis as markers of disease progression.

## 4. Concluding Remarks

**Figure 1:**
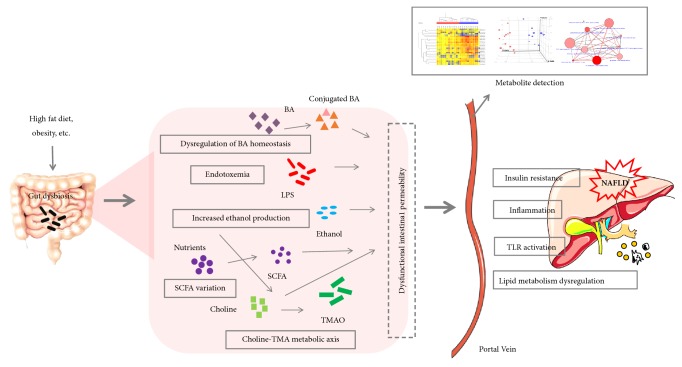
A schematic figure of the role of gut dysbiosis in the development and progression of nonalcoholic fatty liver disease (NAFLD) on the basis of the gut-liver axis. Environmental factors as obesity, high fat diet, or infection (among others) may induce intestinal dysbiosis and also increased intestinal permeability (malfunction of tight junctions). Substances such as short-chain fatty acids, bile acids, bacterial components, choline, and endogenous ethanol reach the liver and activation of toll-like receptors (TLRs) occurs. This activation induces insulin resistance, hepatic inflammation, lipogenesis, and oxidative stress, inducing NAFLD. BA, bile acids; LPS, lipopolysaccharides; SCFA, short chain fatty acid; TLR, toll-like receptor; TMAO, trimethylamine oxide.

Intestinal dysbiosis can trigger intestinal inflammation and increase permeability of the gut epithelial barrier, exposing the hepatobiliary system to gut-derived mediators of dysbiosis, such as bacterial components or metabolites, which may induce NAFLD progression. Gut-derived mediators of dysbiosis contribute to steatosis activate the immune system, induce inflammatory and oxidative pathways, enhance inflammation, and promote fibrogenesis (Figure 1).

Despite the evident association between gut dysbiosis and obesity and NAFLD, derived from experimental studies, very few studies have been conducted in patients with NAFLD in order to explore the role of gut-microbiota derived mediators of dysbiosis in the occurrence and progression of the disease. Most of the previous evidence has been focused on gut microbiota as a therapeutic target to prevent or to treat NAFLD, interfering in gut dysbiosis with probiotic, prebiotic, and symbiotic supplements. Nevertheless, few studies have been focused in gut-derived mediators of dysbiosis as noninvasive markers of disease progression. The study of specific gut-derived mediators of dysbiosis—bacterial components and metabolites—may provide an opportunity to develop a specific diagnostic biomarker for NAFLD. In this sense, we propose the metabolomic study of these and other metabolites involved, in order to achieve a metabolomic profile that could be used as biomarkers for evaluating the status of NAFLD.

## Figures and Tables

**Table 1:** Gut microbiota-derived mediators in human NAFLD.

**Type of metabolites**	**Subjects**	**Type of sample**	**Alterations of gut microbiota-derived mediators**	**References**
SHORT CHAIN FATTY ACIDS	Adults, obesity, NAFLD (SS or NASH)	Blood	Higher abundances of enzymes associated with lactate, acetate, and formate in mild/moderate NAFLD. Higher abundances of enzymes for butyrate, D-lactate, propionate, and succinate in advanced fibrosis	[33]
	Children, obesity, NAFLD	Fecal specimens	Lower acetate, formate, valerate in NAFLD	[34]

BILE ACIDS	Adults, NASH	Blood,Urine	More hydrophobic bile acid profile	[35]
	Adults NAFLD, NASH	Liver	Elevated deoxycholic, chenodeoxycholic, and cholic acids	[36]
	Adults, NAFLD, NASH	Blood	Higher glycocholate, taurocholate, glycochenodeoxycholate in NAFLD	[37]
	Adults, NASH	Fecal specimens	Higher primary to secondary BA ratio in NASH	[38]
	Children NAFLD	Blood	Higher CDCA, unconjugated primary BAs (CDCA + cholic acid), lower DCA, TDCA, GDCA, total DCA, GLCA and total lithocholic acid in NASH	[39]

TLR	Adults, NASH	Blood	Higher TLR-4/MD-2 expression on CD14 positive cells in NASH	[40]

ENDOGENOUS ETHANOL	Children, obesity, NASH	Blood	Elevated blood-ethanol concentration in NASH	[41]
	Children, NAFLD	Blood	Higher ethanol levels in NAFLD	[42]
	Children, obesity, fatty liver	Blood	Higher ethanol levels in NAFLD	[43]

CHOLINE, TMA, TMAO	Children, adolescents, adults	Blood	Decreased choline intake in postmenopausal NAFLD women with fibrosis	[44]
	Adults	Blood	Association of TMAO level and presence/ severity of NAFLD	[45]
	Adults	Blood	Higher free choline levels in NASH	[46]
